# Supplementary material for: Epidemiology and Burden of Sepsis at Thailand’s Largest University-Based National Tertiary Referral Center during 2019
Source: Antibiotics (Basel). 2022 Jul 5;11(7):899. doi: 10.3390/antibiotics11070899 (PMC9312064; doi:10.3390/antibiotics11070899)
Supplement: Supplementary file 1 [file antibiotics-11-00899-s001.zip › Table S1.pdf]

Table S1. Type, frequency, and percentage of 602 causative pathogens detected from 435 patients with infection

**Type of pathogen**	**n (%)**
Bacteria	**532 (88.4)**
**Gram positive bacteria**	141 (23.4)
Staphylococcus aureus	55 (9.1)
Enterococcus faecium	19 (3.2)
Coagulase-negative staphylococci	15 (2.5)
Enterococcus faecalis	14 (2.3)
Streptococcus agalactiae	9 (1.5)
Alpha-hemolytic streptococci	6 (1.0)
Streptococcus pneumoniae	4 (0.7)
Beta-hemolytic streptococci	4 (0.7)
Bacillus spp	2 (0.3)
Nocadia spp.	2 (0.3)
Coryneform bacteria	2 (0.3)
Streptococcus pyogenes	1 (0.2)
Streptococcus infantarius	1 (0.2)
Streptococcus pasteurianus	1 (0.2)
Enterococcus spp.	1 (0.2)
Enterococcus casseliflavus	1 (0.2)
Lactococcus spp.	1 (0.2)
Listeria monocytogenes	1 (0.2)
Corynebacterium spp.	1 (0.2)
Rothia spp.	1 (0.2)
**Gram negative bacteria**	374 (62.1)
Escherichia coli	104 (17.3)
Klebsiella pneumoniae	74 (12.3)
Pseudomonas aeruginosa	61 (10.1)
Acinetobacter baumannii	60 (10.0)
Stenotrophomonas maltophilia	23 (3.8)
Enterobacter spp.	11 (1.8)
Proteus spp.	8 (1.3)
Moraxella spp.	5 (0.8)

Citrobacter spp.	5 (0.8)
Salmonella spp.	4 (0.7)
Burkholderia cepacia	3 (0.5)
Aeromonas spp.	2 (0.3)
Treponema pallidum	2 (0.3)
Fusobacterium spp.	2 (0.3)
Haemophilus influenzae	2 (0.3)
Burkholderia pseudomallei	2 (0.3)
Legionella spp.	1 (0.2)
Klebsiella aerogenes	1 (0.2)
Helicobacter spp.	1 (0.2)
Morganella morganii	1 (0.2)
Brucella spp.	1 (0.2)
Cronobacter sakazakii	1 (0.2)
**Mycobacteria**	17 (2.8)
Mycobacterium tuberculosis complex	14 (2.3)
Non-tuberculous mycobacteria	3 (0.5)
Fungi	**40 (6.6)**
Candida spp.	22 (3.7)
Aspergillus spp.	15 (2.5)
Cryptococcus spp.	1 (0.2)
Pneumocystis jirovecii	1 (0.2)
Trichosporon spp.	1 (0.2)
Virus	**30 (5.0)**
Influenza/Respiratory syncytial virus	15 (2.5)
Dengue virus	5 (0.8)
Cytomegalovirus	4 (0.7)
Parainfluenza virus	2 (0.3)
Human metapneumovirus	2 (0.2)
Chikungunya virus	1 (0.2)
Adenovirus	1 (0.2)
**Total causative pathogens detected**	**602 (100.0)**
