# Supplementary figures and images for: Epidemiology and Burden of Sepsis at Thailand’s Largest University-Based National Tertiary Referral Center during 2019
Source: Antibiotics (Basel). 2022 Jul 5;11(7):899. doi: 10.3390/antibiotics11070899 (PMC9312064; doi:10.3390/antibiotics11070899)

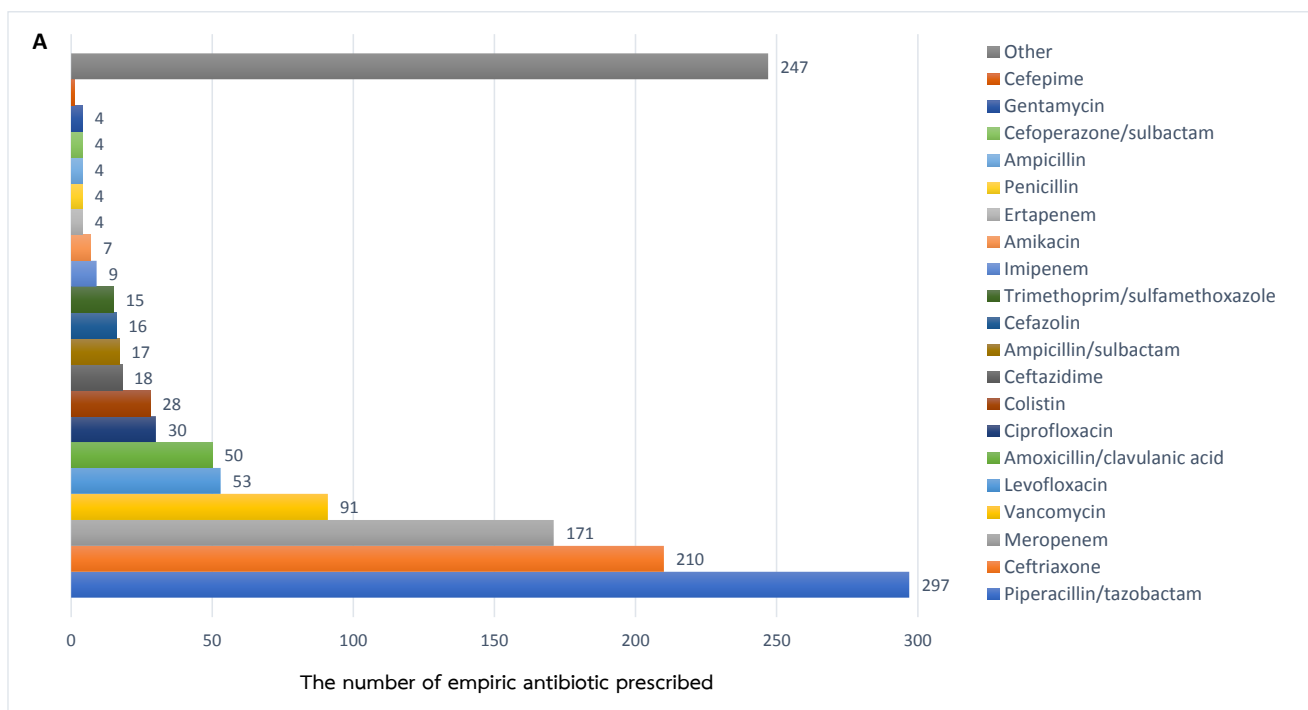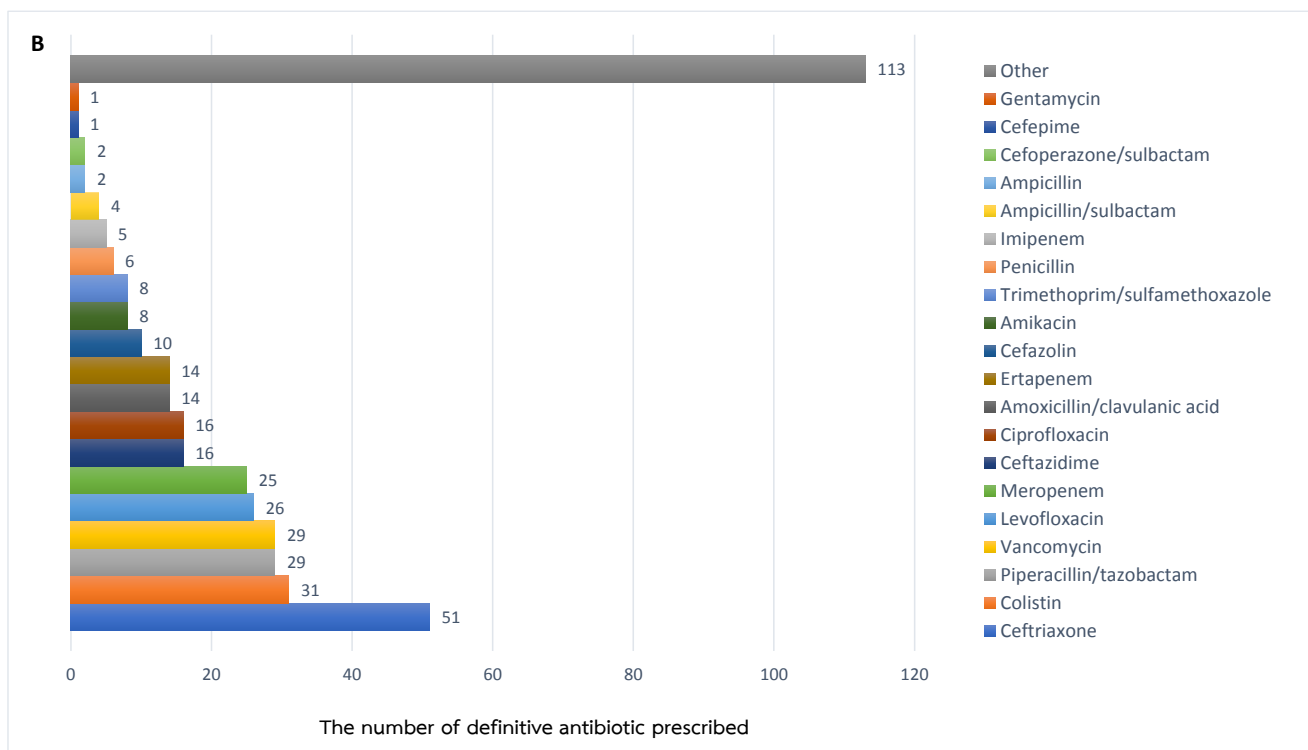

Supplement: Supplementary file 1 [file antibiotics-11-00899-s001.zip › Figure S1.pdf]
